# Dissecting Out Differences in the Transcriptomes of Inflammatory Bowel Disease

**DOI:** 10.1371/journal.pmed.0020273

**Published:** 2005-08-23

Inflammatory bowel disease (IBD) is the term that encompasses chronic relapsing diseases characterized by inflammation of the bowel, specifically ulcerative colitis (UC) and Crohn disease (CD). They are common disorders; in the UK, for example, together they affect about one person in every 400, amounting to a total of 120,000 cases of UC and 60,000 of CD, with 6,000 new cases of UC and 3,000 new cases of CD every year. Current estimates total at more than 1 million cases in the US and Europe. The symptoms for both these conditions—which suggest an abnormal immune response at the intestinal mucosa—include abdominal pain, diarrhea, fever, severe fatigue, and weight loss. Current understanding of disease pathogenesis suggests a complex action of multiple environmental factors that trigger disease in individuals with a susceptible genetic background.

Today, finding genes that have a role in diseases has been made easier by the sequencing of the human genome and creation of an expressed sequence tag clone database. Previous positional cloning studies have revealed three genes associated with IBD that carry variants with a causative role. Another of the tools for analyzing genes is microarray technology, in which the expression of transcripts of thousands of genes can be investigated simultaneously. This approach offers an insight into disease pathophysiology.

In a research article published in *PLoS Medicine*, Christine Costello and colleagues have gone further with cDNA microarrays to attempt to decipher gene regulatory events and identify genes that might be involved in the pathophysiology of these IBD. They found 650 genes that were differentially regulated between normal control individuals and the individuals with one of the two IBD subtypes. In fact, 500 and 272 differentially regulated transcripts were identified between control individuals and patients with CD and UC, respectively. There was an imbalance between over- and underexpressed genes in the IBD subtypes. In CD, approximately 84% of differentially expressed genes were found to be down-regulated compared with 42% of genes in UC. However, the authors caution that this finding was highly influenced by the types and numbers of genes present on any microarray system; in addition, none of the 122 differentially expressed genes in CD and UC was overexpressed in one disease and underexpressed in the other. These observations support the notion of a shared general inflammatory profile underlying each form of IBD, with more specific events in the pathophysiological cascade being disease-specific.[Fig pmed-0020273-g001]

**Figure pmed-0020273-g001:**
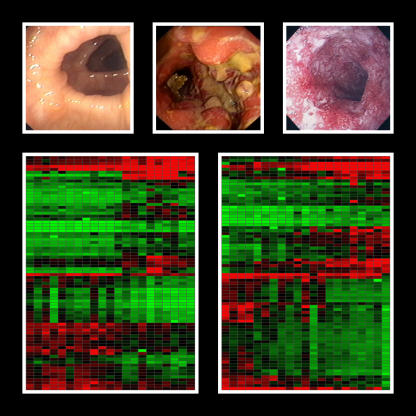
Colonoscopy images of normal control, Crohn Disease (CD) and ulcerative colitis (UC) patients (top, left to right). Differentially expressed genes in controls vs. CD (lower left) and controls vs. UC patients (lower right)

In a second part of the study, the team tried to interpret the functional consequences of changes in gene expression observed in the microarray analysis. They used an annotation-based pathway database, which classified differentially expressed genes into three major groups: immune and inflammatory response; oncogenesis, cell proliferation, and growth; or structure and permeability. For immune and inflammatory response, the team identified many genes associated with aberrant immune response; it is not surprising, perhaps, to find a general up-regulation of immune response and antigen presentation in IBD. Several genes associated with cell growth and proliferation were up-regulated in UC—a finding similar to previous microarray studies that had reported involvement of cancer-related genes in IBD (although the altered genes were different). There was also an enrichment of genes associated with structure and permeability; in this class several genes were ubiquitously altered in both IBD and non-IBD samples, reflecting dysregulation of genes for paracellular permeability, degradation of extracellular matrix, and barrier protection against bacterial invasion of the epithelial surface.

Ultimately this study highlights the complex pathogenesis of UC and CD, and indicates some possible future avenues for research of mucosal diseases in general. It demonstrates that genomic technologies are suitable to directly dissect human pathophysiology.

